# Sphingolipids in Inflammation

**DOI:** 10.1155/2018/7464702

**Published:** 2018-05-06

**Authors:** Elisabetta Albi, Alice Alessenko, Sabine Grösch

**Affiliations:** ^1^Department of Pharmaceutical Science, University of Perugia, Perugia, Italy; ^2^Russian Academy of Sciences, Moscow, Russia; ^3^Institute of Clinical Pharmacology, Frankfurt, Germany

The etiology of many diseases results from the dysregulation of inflammation. Understanding the molecular mechanisms controlling the inflammatory response is essential to formulate therapeutic strategies for the treatment of inflammatory conditions. In fact, substantial research has unveiled important aspects of the inflammatory machinery, both at the cellular and molecular levels. Recently, sphingolipids (Sph) have emerged as signaling molecules that regulate many cell functions, and ample evidence emphasizes their role in the regulation of inflammatory responses.

This special issue addresses the role of different Sph as mediators of inflammation and/or of oncogenic inflammatory signaling. The topics provide information about the behavior of enzymes for sphingolipid metabolism and sphingolipid metabolites *in vitro* in various experimental cell models as well as *in vivo* in animal and patient blood. The issue includes thirteen papers, six reviews, and seven research articles. The papers highlight the following aspects: the action of Sph in various phases of acute inflammatory response, the behavior of enzymes that produces ceramides (Cer), those that utilize Cer to produce other bioactive molecules such as Cer-1-phosphate (Cer1P) and sphingosine-1-phosphate (S1P), and those that transform S1P such as S1P phosphatase and S1P lyase in inflammation and cancer.

The different sphingolipids mediate their cellular effects both as components of cellular membranes as well as they act as signaling molecules in the cell. Membrane-bound sphingolipids can be organized in so-called lipid rafts or detergent-resistant membranes (DRMs). These temporal and spatial restricted membrane areas are signaling platforms for different membrane receptors. Instead, Cer, Cer1P, sphingosine (S), and S1P play the role of second messengers in transduction of signals from various external stimuli by binding to their respective receptors or/and by interacting with intracellular proteins. In this regard, the first review of this special issue written by S. Grösch et al. “The Many Facets of Sphingolipids in the Specific Phases of Acute Inflammatory Response” summarizes the cellular effects of different sphingolipids in the various phases of inflammation. We addressed the role of sphingolipids in cell migration, recognition of exogenous agents, and in activation/differentiation of immune cells. The second paper presents original research data focusing on the role of DRMs in the pathophysiology of cystic fibrosis. In the research article “Evidence for the Involvement of Lipid Rafts and Plasma Membrane Sphingolipid Hydrolases in *Pseudomonas aeruginosa* Infection of Cystic Fibrosis Bronchial Epithelial Cells,” D. Schiumarini et al. show that the infection of cystic fibrosis bronchial epithelial cells with *Pseudomonas aeruginosa* is dependent on the formation of such DRMs. Further, they demonstrate that GBA1 and SMase activities are more than double increased in these DRMs which contribute to an enhanced IL-8 production in these cells.

The neutral sphingomyelinase (nSMase) plays also an important role in the pathophysiology of Parkinson's disease. In the research paper “Neutral Sphingomyelinase Behaviour in Hippocampus Neuroinflammation of MPTP-Induced Mouse Model of Parkinson's Disease and in Embryonic Hippocampal Cells,” Cataldi S. et al. show that in the 1-methyl-4-phenyl-1,2,3,6- tetrahydropyridine- (MPTP-) induced mouse model of Parkinson disease, the nSMase is downregulated. Treatment of embryonic hippocampus cultured cells with vitamin D3 increased nSMase activity leading to a significant decrease in the levels of saturated SM species and a significant increase of unsaturated SM species. These changes might determine enhanced dynamic properties of the cells contributing to cell differentiation.

The ceramide produced by nSMase is phosphorylated by Cer kinase (CerK) to form Cer-1-phosphate (C1P). In the paper entitled “Implication of Ceramide Kinase in Adipogenesis,” Ordoñez et al. demonstrate the ability of CerK to regulate adipocyte differentiation associated with obesity. CerK gene silenced by siRNA is responsible for the structure and function changes of adipocytes as the reduction of lipid droplet formation, depletion of triacylglycerols, block of leptin secretion, and downregulation of peroxisome proliferator-activated receptor gamma, suggesting that targeting CerK might be an innovative therapeutic strategy for obesity.

A link between inflammation and carcinogenesis has been appreciated for over a century. Apart from cytokines and chemokines, lipid mediators, particularly C1P and S1P, contribute to inflammation and cancer. S1P is an important player in inflammation-associated colon cancer progression. On the other hand, C1P has been recognized to be involved in cancer cell growth, migration, survival, and inflammation. In N. C. Hait and A. Maiti's review paper, the authors presented “The Role of Sphingosine-1-Phosphate and Ceramide-1-Phosphate in Inflammation and Cancer.” The review demonstrates a role of S1P as a biomarker for cancer progression after measuring the blood levels in human subjects. Plasma S1P levels in ovarian cancer patients were almost twice as high as in healthy controls. Few studies have demonstrated that CerK activation and intracellular C1P are involved in noncancer and cancer cell growth and survival. CerK has also been found to be overexpressed in breast cancer and associated with poor prognosis. CerK promotes tumor cell survival and mammary tumor recurrence. Originally, CerK/C1P has been shown to enhance lung cancer cell growth and survival. It has been shown that CerK/C1P is involved in pancreatic cancer cell migration, invasion, and survival.

A multitude of the immunomodulatory effects of S1P have been attributed to signaling through S1PR1, whereas the contribution of other S1P receptors remains largely obscure. S1PR4 is particularly expressed by immune cells and may therefore be critically involved in immunomodulation by S1P. In C. Olesch's review “Beyond Immune Cell Migration: The Emerging Role of the Sphingosine-1-Phosphate Receptor S1PR4 as a Modulator of Innate Immune Cell Activation,” it summarized the current knowledge about S1PR4 and discussed therapeutic implications of interfering with its signaling, particularly in chronic inflammatory disease settings. S. N. Syed et al. in the review “S1P Provokes Tumor Lymphangiogenesis via Macrophage-Derived Mediators Such as IL-1β or Lipocalin-2” highlight the role of S1P derived from tumor apoptotic cells in changing the tumor-associated macrophage phenotype to promote lymphangiogenesis. In the “Effects of FTY720 on lung injury induced by hindlimb ischemia reperfusion in rats,” L. Wang et al. demonstrate that the use of a structural analogue of S1P (*FTY720*) attenuates lung injury induced by ischemia-reperfusion by modulating S1P lyase (S1PL), sphingosine kinase 1 (SphK1), and SphK2.

The next two papers deal with the role of S1P in breast cancer. The first paper written by J. Tsuchida et al. with the title “Clinical Impact of Sphingosine-1-Phosphate in Breast Cancer” summarizes the current knowledge about S1P in breast cancer progression, the clinical impact of S1P in human breast cancer patients, and S1P as therapeutic target for breast cancer patients and its role in chemotherapy resistance. The second paper is a research paper investigating plasma S1P level in breast cancer patients with the title “Paradoxical Association of Postoperative Plasma Sphingosine-1-Phosphate with Breast Cancer Aggressiveness and Chemotherapy” by R. Ramanathan et al. Here, plasma S1P level of breast cancer patients and healthy volunteers were compared as well as the changes of plasma S1P level during and after chemotherapy detected.

It has been postulated that S1P levels inside cells are tightly regulated by the balance between the S1P synthesizing enzymes Sphk1/2 and by metabolizing/degrading enzymes like phosphatases and S1P lyase (SGPL-1). SGPL-1 is thereby irreversibly degrading S1P into hexadecenal and phosphoethanolamine. S1P phosphatases 1 and 2 (SGPP-1/2) reversibly metabolize S1P into sphingosine. In A. Schwiebs et al.'s paper “Nuclear Translocation of SGPP-1 and Decrease of SGPL-1 Activity Contribute to Sphingolipid Rheostat Regulation of Inflammatory Dendritic Cells,” the authors showed that the major proportion of endogenous SGPP-1 is located in the nuclear compartment in murine and human differentiated dendritic cells. Upon inflammatory stimuli, translocation of SGPP-1 into the ER occurs and potentially contributes to S1P fate and sphingolipid rheostat in dendritic cell immune response. Spns2 is only marginally expressed in dendritic cells and is rather not relevant for S1P transportation in inflammatory dendritic cell. Authors conclude that the systematic shift of SGPP-1 from nucleus to cytoplasm contributes to the metabolism of S1P into sphingosine and thus loss of cytoplasmic S1P. Current investigation demonstrates quite complex sequence of S1P and sphingosine rheostat with corresponding sphingolipid enzyme and transporter expression.

Also, the next paper “S1P Lyase Regulation of Thymic Egress and Oncogenic Inflammatory Signaling” by A. Kumar and coauthors deals with the role of S1P lyase in oncogenic inflammation. Cleavage of S1P by SPL represents the final step in the sphingolipid catabolic pathway. Primarily through its control of intracellular S1P levels and extracellular S1P gradients, SPL regulates lymphocyte trafficking, inflammation, and other physiological and pathological processes. For example, SPL located in thymic dendritic cells acts as a metabolic gatekeeper that controls the normal egress of mature T lymphocytes from thymus into the circulation, whereas SPL deficiency in gut epithelial cells promotes colitis and colitis-associated carcinogenesis (CAC). Authors showed that they identified a complex syndrome comprised of nephrosis, adrenal insufficiency, and immunological defects caused by inherited mutations in human SGPL1, the gene encoding SPL. Authors also summarize important new insights regarding S1P metabolism, SPL structure, and recently recognized functions of SPL in the context of embryonic development and the pathophysiology of human disease. It discussed progress in the development of SPL inhibitors and their potential therapeutic applications.

At the end, this special issue gives an overview about the current knowledge of different sphingolipids in the many processes of inflammation and gives also suggestions regarding future experiments to explain clinical data demonstrating changes in sphingolipid level in inflammatory processes.

*Elisabetta Albi* *Alice Alessenko* *Sabine Grösch*

